# Development and baseline findings of a national dataset describing Australian private practice dietetics

**DOI:** 10.1111/1747-0080.70027

**Published:** 2025-08-06

**Authors:** Amy Kirkegaard, Olivia Wright, Peter Clark, Lauren Ball

**Affiliations:** ^1^ Centre for Community Health and Wellbeing The University of Queensland Springfield Queensland Australia; ^2^ School of Human Movement and Nutrition Sciences The University of Queensland Springfield Queensland Australia; ^3^ Faculty of Health Sciences and Medicine Bond University Robina Queensland Australia; ^4^ School of Public Health The University of Queensland St Lucia Queensland Australia

**Keywords:** dietetics, private practice, small business, workforce

## Abstract

**Aims:**

This study aimed to establish the processes for a national dataset describing Australian private practice dietetics and to report baseline workforce data.

**Methods:**

A cross‐sectional survey captured workforce and business data for dietitians working in private practice dietetics in Australia. Business owners from a national register were invited to complete the survey and to share the survey with employed or contracted dietitians between May and June 2024. Data were analysed descriptively.

**Results:**

Seven hundred and three business owners were invited to participate, and 176 participants completed the workforce items (25% response rate). Participants were primarily female (*n* = 158; 90%) business owners (*n* = 161; 91%), mean ± SD age of 42 ± 9 years and mean time since graduation of 15 ± 9 years. Mean time spent per week working in private practice was 26 ± 15 hours. Most participants were satisfied with their work in private practice (*n* = 160, 91%) and anticipated working in private practice in 5 years' time (*n* = 155, 88%), though some (*n* = 62, 35%) felt some degree of unwellness due to stress. Business owners were generally dissatisfied with the applications received for advertised dietetics roles. One hundred twenty‐one participants consented to follow‐up in 2 years as part of the ongoing survey.

**Conclusions:**

A national dataset describing the dietetics workforce, supported by ongoing data capture, is essential to support workforce planning and ensure that private practice is a legitimate and sustainable career path, thereby future‐proofing this segment of the workforce and ensuring access to high‐quality preventative and primary dietetics care.

## 1 INTRODUCTION

Chronic diseases are the leading causes of morbidity and mortality in Australia,^1^ with both *Australia's Long Term National Health Plan* and *Australia's Primary Health Care 10 Year Plan 2022–2032* describing chronic disease as a significant challenge facing the Australian health system.^2^, ^3^ Diet is the third highest modifiable risk factor for disease burden,^1^ and when combined with evidence supporting the effectiveness of dietetic care in treating and managing chronic conditions,^4^ identifies a clear role for dietitians in addressing chronic disease in Australia. To this end, a highly skilled and geographically distributed workforce is essential to ensuring access to appropriate support to prevent and manage chronic disease.^2^, ^3^

Private practice is a growing area of practice for Australian dietitians, with an increasing proportion of dietitians and dietetics graduates finding employment in this area of practice.^5^, ^6^, ^7^, ^8^ The introduction of Medicare Benefits Schedule items for dietetic services in 2004 has been described as a significant contributor to the expansion of private practice dietetics in Australia,^8^ with dietetic items accruing benefits totaling AU$31 million in 2024.^9^ Dietetics has consistently been among the top five most utilized allied health chronic disease management services, and the introduction of items for eating disorders in 2019 further enhanced access to dietetic care.^9^

Dietitians working in private practice have identified knowledge and skills gaps, describing universities as being slow to adapt and respond to new areas of practice resulting in a limited focus on private practice during pre‐credentialling education.^10^, ^11^, ^12^ Such capability gaps may fail to adequately prepare dietitians to navigate the constantly changing environment of private practice, which spans political, legal and economic influences, to changes in technology and food systems.^13^ Most recently, the Scope of Practice Review recommended a suite of changes to redesign primary care, one of which was making participation in practice accreditation mandatory.^14^ While practice accreditation schemes aim to assure safety and quality through implementation of standards, their success is contingent on the knowledge and attitudes of individuals responsible for their implementation in practice.^15^ Private practice dietitians have previously identified business acumen as a clear capability gap that is often overcome through experience,^12^ and may impede participation in accreditation if dietitians are not supported appropriately. Workforce data is essential to understanding these capability gaps and informing workforce planning.

Given the potential role dietitians could play in reducing chronic disease in Australia, and the significant and growing proportion of Australian dietitians working in private practice, comprehensive and timely workforce and business data is essential to inform workforce planning to support sustainable, high‐quality care.^14^, ^16^ However, limited workforce data have long been an issue for Australian dietitians.^5^ The most recent peer‐reviewed study comprehensively describing the private practice dietetic workforce was conducted by Ball et al. in 2013.^17^ Considering this study was published over a decade ago, there is a significant gap regarding the current private practice workforce. Such a gap constrains support and advocacy efforts, sustaining challenges inherent in private practice and promoting workforce attrition. Additionally, such data could identify system efficiencies and provide guidance on quality improvements and more effective resource utilisation.^18^ Therefore, this study aimed to establish the processes and baseline workforce data of a national survey of Australian dietitians working in private practice. The survey will provide a vehicle for workforce and business data to be captured in a regular and meaningful manner from this evolving area of practice, becoming an asset to the profession.

## 2 METHODS

From a pragmatic philosophical positioning, we developed and tested a cross‐sectional, online survey to capture data describing the workforce and businesses that, together, comprise private practice dietetics in Australia. The survey structure was developed by the research team, drawing on concepts from Cant,^19^, ^20^ Ball,^17^ and the RACGP Health of the Nation 2023 report.^21^ However, in contrast to these publications, the survey aimed to capture data at two levels—individual and business—to describe both dietitians and the practices in which they are employed. A ‘private practice’ was defined using the Miriam‐Webster dictionary as ‘a professional business that is not controlled or paid for by the government or larger organisation (such as a hospital)’.^22^ This broad definition allowed inclusion of traditional private practices that delivered dietetic consultations as well as practices that offered innovative services or products, including programmes, applications and tools, and business coaching. The final survey was conducted using Qualtrics,^23^ and included items relating to workforce (e.g., employment status and settings, activities performed, and time investment) and business (e.g., business ownership, size, and operation, team composition and remuneration, and products and services).

The survey was pretested in January and February 2024 with seven private practice dietitians (‘testing dietitians’) representing a range of practices differing in size and scope. Pretesting was conducted via Microsoft Teams as this allowed the researcher to observe the testing dietitian and their screen as they completed the survey, noting observations for discussion in the debriefing interview.^24^ Once the survey was completed, the lead researcher debriefed with the testing dietitian to gather feedback on survey items, design, and process. Data generated from the pretest survey were not included in the final survey results. Early pretesting resulted in structural changes to the survey, whereby branch logic was introduced to ensure respondents were only presented with questions relevant to their circumstances, thereby minimising respondent burden. This required the survey to be divided into two sections. The first section included questions about dietitians working in private practice in any capacity, while the second section included questions about the associated business. Later pretesting focused on the clarity and comprehension of item wording and language.

To increase the likelihood that all dietitians working in private practice were invited to participate in the study, a register of private practices was developed to provide a comprehensive list of verified dietetics businesses for invitation to the study. Publicly available data for dietitians registered on the ‘Find a Dietitian’ database administered by Dietitians Australia were obtained. To identify private practices and business owners, each entry was reviewed by entering the business name (or dietitian name and ‘dietitian’) into the Google search engine. Where a business website existed, the website was reviewed to verify the business owner and contact information, which were updated in the register, if required. Where a business website did not exist, other websites were reviewed to verify that the dietitian was currently practising, including general practice websites and other dietitian directories (e.g., FODMAP Dietitians Directory, ANZAED Find Eating Disorder Help) and LinkedIn. Additional dietetics businesses that were identified during this process were added to the register. The final register included 703 dietetics practices for which an owner and contact details could be verified.

Between May and June 2024, invitations were e‐mailed to all listed owners on the register of dietetics practices. The first e‐mail introduced the survey and provided the participant information sheet. Business owners were invited to participate via a link to the survey contained in the e‐mail. A second e‐mail was sent approximately 2 weeks later and reiterated eligibility criteria, highlighting that eligibility extended to businesses offering non‐traditional products or services. Reminder e‐mails were sent 1 week, 3 days, and 1 day before the survey closed. All e‐mails included the request that business owners share the survey with dietitians employed or contracted by their business so that they could complete the workforce section of the survey. Consent was obtained electronically on the first page of the survey. Business owners who completed the survey could opt to receive a report summarising survey responses for use as a business benchmarking tool once the survey closed. The use of benchmarking data has been reported by others to increase survey participation.^25^ The report was compiled by the research team after the survey closed and disseminated to participants who opted to receive the report in September 2024.

The survey was anticipated to be conducted every 2 years, acknowledging the need to balance the currency of data with the burden placed on dietitians completing the survey; however, there is an opportunity for annual administration if the survey were to be integrated into the professional membership process. The register of dietetics businesses is a living document that will be updated with newly identified private practices, with information sourced from the ‘Find a Dietitian’ search tool and by cross referencing other dietitian directories (FODMAP Dietitians Directory, ANZAED Find Eating Disorder Help, Yellow Pages) and results from the Google search engine. Business owners will be invited to register their interest in ongoing participation in the survey, with those agreeing to do so allocated a unique identifier to allow data linking across subsequent surveys. Commitment to ongoing participation will include sharing surveys with employed and contracted dietitians; we anticipate that formal registration in the survey will enhance workforce participation in subsequent surveys. Data captured in subsequent rounds will be linked and compared to provide new insights into how practices evolve over time.

Data describing dietetics businesses are reported elsewhere.^26^ Data describing the dietetics workforce were analysed using IBM SPSS Statistics for Windows (version 29.0.2.0; Armonk, NY: IBM Corp).^27^ The representativeness of the sample was determined using chi‐square goodness‐of‐fit analyses for location, gender and age against membership data from Dietitians Australia as at December 2024. Age, time since graduation, and time spent on activities were captured as continuous variables but transformed into ordinal variables for analysis. Frequencies and percentages were calculated for categorical variables. Statistical tests were applied to investigate the strength and direction of association between two variables. Specifically, Kendall's Tau‐*b* test was used to investigate differences between categorical variables, such as time spent on client and business activities and satisfaction; see Table S1 for variables tested. Short answer responses were analysed by the lead author using content analysis, whereby responses were reviewed and categories describing the data generated and discussed among the research team.^28^ Frequencies were quantified by summing the number of individual responses for each category.^28^ Themes related to the dietetics workforce are reported here.

Ethical approval for this study was obtained from the UQ Human Research Ethics Committee (2023/HE001399). This study was reported in accordance with the Strengthening the Reporting of Observational Studies in Epidemiology Statement.^29^

## 3 RESULTS

Over seven hundred (*n* = 703) business owners operating a private dietetics practice were invited to participate in the survey. One hundred seventy‐six participants completed the workforce section (25% response rate), with 154 participants also completing the business section. Dietitian participants were primarily female (*n* = 158; 90%) with a mean age of 42 years (SD 9; range 25–75 years). Most dietitians were business owners (*n* = 161; 91%) and all were APDs (*n* = 176; 100%). The mean time since graduation was 15 years (SD 9; range 2–43 years). No significant differences were observed between survey respondents and Dietitians Australia membership in terms of gender (*χ* ^2^ = 2.90; *p* = 0.23), or location (*χ* ^2^ = 6.33; *p* = 0.39); however, a significant difference was observed in terms of age (*χ* ^2^ = 48.89; *p* = <0.001), with respondents being older than Dietitians Australia members. Demographic characteristics are presented in Table 1.

**TABLE 1:** Demographic characteristics of participants who were working as a dietitian in private practice as an employee, contractor, or business owner (*n* = 176).

Characteristic	*n* (%)
Gender	
Female	158 (90)
Male	16 (9)
Non binary	1 (<1)
Prefer not to say	1 (<1)
Age	
21–30 years	22 (13)
31–40 years	64 (36)
41–50 years	57 (32)
51–60 years	29 (16)
>60 years	4 (2)
Time since graduation	
<5 years	17 (10)
5–10 years	51 (29)
11–20 years	60 (34)
21–30 years	37 (21)
31–40 years	10 (6)
>40 years	1 (<1)
APD status	
Provisional APD	1 (<1)
APD	169 (96)
Advanced APD	5 (3)
Fellow of Dietitians Australia	1 (<1)
Employment status	
Employee only	10 (6)
Contractor only	5 (3)
Business owner only	158 (90)
Employee and owner	1 (<1)
Contractor and owner	1 (<1)
Employee, contractor, and owner	1 (<1)

Most participants were business owners that operated a single business (*n* = 149; 93%); however, a smaller number owned two (*n* = 7; 4%), three (*n* = 2; 1%), five (*n* = 1; <1%) or seven (*n* = 1; <1%) businesses. A small number of business owners were also employed (*n* = 1; <1%), contracted (*n* = 1; <1%), or both employed and contracted (*n* = 1; <1%) by another private practice, though more were employed in dietetics work outside of the private practice sector (*n* = 67; 42%), or in non‐dietetic work (*n* = 30; 19%). Most participants who identified that they were solely employed in private practice worked at a single practice (*n* = 9; 82%), with the remaining participants (*n* = 2; 18%) employed by two private practices. In contrast, participants who were contracted by a practice worked at one (*n* = 5; 63%) or two (*n* = 3; 37%) practices. All employees and contractors were involved in client‐related activities. Client‐related activities related to care for individual clients and included care facilitation (e.g., corresponding with a client's healthcare team, reviewing a client's health information before a consultation) and delivery (e.g., delivering consultations or programmes). However, employees were also involved in other activities, including quality improvement (*n* = 7; 70%), service design (*n* = 6; 60%), business development (*n* = 4; 40%), staff management (*n* = 3; 30%), and product design (*n* = 2; 20%).

Time spent by dietitian participants in various activities across client‐ and business‐related activities is presented in Table 2. The mean time spent working in private practice was 26 hours per week (SD 15 hours; range 0–80 hours). Time in paid dietetics‐related work outside of private practice was primarily spent in hospital (*n* = 26), research (*n* = 17), community and public health (*n* = 16), and tertiary education (*n* = 9). Twenty‐five participants identified other dietetics‐related areas of work including mentoring and supervision, sporting and aged care organisations, and advisory roles. Time in paid work not related to dietetics was primarily spent in research (*n* = 4), fitness (n = 4), health care (*n* = 2) and retail (*n* = 2). Nineteen participants engaged in non‐dietetics‐related work in a diverse range of fields, including exercise science or physiology, support work, sports coaching, administration, accounting and bookkeeping, practice management, farming, and property and accommodation. There were no statistically significant associations between time spent on client and business activities and satisfaction with work in private practice (*p* > 0.05).

**TABLE 2:** Proportion of time spent per week by dietitians participating in this study working across various roles and settings (*n* = 176).

Characteristic	*n* (%)
Time spent on client‐related activities^a^	
0 h	6 (3)
1–10 h	55 (31)
11–20 h	76 (43)
21–30 h	20 (11)
31–40 h	15 (9)
41–50 h	2 (1)
>50 h	1 (<1)
Time spent on business‐related activities^a^	
0 h	5 (3)
1–10 h	118 (67)
11–20 h	30 (17)
21–30 h	13 (7)
31–40 h	7 (4)
41–50 h	2 (1)
Time spent in other paid dietetics‐related work	
0 h	100 (57)
1–10 h	31 (18)
11–20 h	20 (11)
21–30 h	15 (9)
31–40 h	9 (5)
41–50 h	1 (<1)
Time spent in other paid work	
0 h	145 (82)
1–10 h	21 (12)
11–20 h	6 (3)
21–30 h	3 (2)
31–40 h	1 (<1)

^a^ *n* = 175 as data was missing for one participant.

Content analysis of short answer responses about challenges experienced in private practice identified the challenge of balancing paid (i.e., client‐facing tasks) with unpaid tasks (*n* = 12), which included (in order of number of times mentioned) corresponding with other health professionals or supports (*n* = 19), business administration tasks (*n* = 13), reception tasks (e.g., booking appointments, taking payments) (*n* = 7), professional development (*n* = 6), marketing and social media (*n* = 4), client resources (*n* = 2), and client‐related tasks before and after consultations (e.g., reviewing referral, writing notes) (n = 2).

Participant satisfaction with their work as dietitians in private practice is presented in Table 3. Significant positive correlations were observed between satisfaction and accomplishment (*τ* = 0.280, *p* < 0.001), remuneration (*τ*_*b*_ = 0.221, *p* < 0.001) and support (*τ*_*b*_ = 0.178, *p* = 0.005), suggesting that as dietitians' sense of accomplishment and perceived adequacy of remuneration and support increases, so too does their satisfaction with their work. A significant positive correlation between years since graduation and support (*τ*_*b*_ = 0.13, *p* = 0.030) was observed, suggesting that perceived adequacy of support increases with years since graduation. Conversely, a negative correlation between satisfaction and unwellness (*τ*_*b*_ = −0.192, *p* = 0.003) was observed, suggesting that as work stress leads dietitians to feel more unwell, their level of satisfaction with their work decreases. In addition, a significant negative relationship between age and unwellness (*τ*_*b*_ = −0.153, *p* = 0.015) was observed, suggesting that as age decreases, level of unwellness increases. Finally, a significant correlation between satisfaction and likelihood of working in private practice in 5 years' time (*τ*_*b*_ = 0.185, *p* = 0.005) was observed. The impact of stress arising from working in private practice on health was described by nine participants in short answer responses.

**TABLE 3:** Satisfaction with employment in private practice of dietitians participating in this study (*n* = 176).

Characteristic	*n* (%)
Sense of accomplishment	
Completely agree	52 (30)
Mostly agree	77 (44)
Somewhat agree	23 (13)
Somewhat disagree	6 (3)
Mostly disagree	9 (5)
Completely disagree	9 (5)
Received adequate remuneration	
Completely agree	18 (10)
Mostly agree	50 (28)
Somewhat agree	45 (26)
Somewhat disagree	32 (18)
Mostly disagree	18 (10)
Completely disagree	13 (7)
Received adequate support	
Completely agree	27 (15)
Mostly agree	38 (22)
Somewhat agree	58 (33)
Somewhat disagree	33 (19)
Mostly disagree	16 (9)
Completely disagree	4 (2)
Felt unwell due to stress	
Completely agree	4 (2)
Mostly agree	12 (7)
Somewhat agree	46 (26)
Somewhat disagree	23 (13)
Mostly disagree	55 (31)
Completely disagree	36 (20)
Satisfaction with work in private practice	
Completely satisfied	70 (40)
Mostly satisfied	63 (36)
Somewhat satisfied	22 (13)
Somewhat dissatisfied	14 (8)
Mostly dissatisfied	5 (3)
Completely dissatisfied	2 (1)
Likelihood of working in private practice in 5 years	
Extremely likely	28 (16)
Likely	95 (54)
Somewhat likely	37 (21)
Somewhat unlikely	12 (7)
Unlikely	4 (2)
Extremely unlikely	0 (0)

Thirty‐seven participants (contractor *n* = 1, employee *n* = 4, owner *n* = 32) had applied for a dietetic job in private practice within the last 12 months. The most important factors that motivated participants to apply for these roles were work hours (*n* = 19; 20%), remuneration (*n* = 15; 16%), location (*n* = 15; 16%), employment arrangement (*n* = 13; 14%), and team environment (*n* = 13; 14%). The least important factors were role responsibilities (*n* = 6; 7%), opportunities for career progression (*n* = 5; 5%), supervision and mentoring (*n* = 3; 3%), and professional development (*n* = 3, 3%). Six participants did not complete the question identifying the most important factors. Poor remuneration (*n* = 3) and lack of understanding about remuneration entitlements (*n* = 1) were raised by participants in short answer responses; however, the need to balance employee expectations in terms of remuneration with business profitability was similarly raised (*n* = 3).

Thirty‐five businesses advertised a total of 64 dietetics jobs in the last 12 months, including positions for dietitians with <2 years' experience (*n* = 18; 28%), 2–<5 years' experience (*n* = 24; 38%), 5–<10 years' experience (*n* = 15; 23%), and 10 or more years' experience (*n* = 7; 11%). Generally, more business owners were dissatisfied with the number and quality of applications for roles across all levels of experience, as illustrated in Table 4. Business owners were most dissatisfied with the number (*n* = 11; 73%) and quality (*n* = 9; 60%) of applications for roles for dietitians with 5–<10 years' experience and least dissatisfied with the number (*n* = 11; 61%) and quality (*n* = 9; 50%) of applications for roles for dietitians with less than 2 years' experience. Associations between level of satisfaction and geographic location and regionality were not analysed due to the small number of responses. Recruitment of suitably experienced staff was identified as a challenge in private practice by participants (*n* = 8), with others expanding on this by describing the disruption of team changes and the financial and time cost of onboarding and training. Retention of dietitians in rural locations (*n* = 2) was also identified as a challenge; however, the greatest challenge in terms of workforce was the general management and supervision of staff (*n* = 11), which included balancing workload with staff support to ensure growth and prevent burnout.

**TABLE 4:** Business owners' satisfaction with the number and quality of applications received for advertised dietetic roles across four experience levels.

Level of satisfaction	Number of applications	Quality of applications
	*n* (%)	
Advertised role for dietitian with <2 years' experience (*n* = 18; 28%)		
Completely satisfied	‐	‐
Mostly satisfied	4 (22)	4 (22)
Somewhat satisfied	3 (17)	5 (28)
Somewhat dissatisfied	3 (17)	2 (11)
Mostly dissatisfied	3 (17)	4 (22)
Completely dissatisfied	5 (28)	3 (17)
Advertised role for dietitian with 2 to <5 years' experience (*n* = 24; 38%)		
Completely satisfied	3 (13)	1 (4)
Mostly satisfied	4 (17)	5 (21)
Somewhat satisfied	2 (8)	5 (21)
Somewhat dissatisfied	2 (8)	1 (4)
Mostly dissatisfied	3 (13)	4 (17)
Completely dissatisfied	10 (42)	8 (33)
Advertised role for dietitian with 5 to <10 years' experience (*n* = 15; 23%)		
Completely satisfied	1 (7)	1 (7)
Mostly satisfied	2 (13)	4 (27)
Somewhat satisfied	1 (7)	1 (7)
Somewhat dissatisfied	2 (13)	2 (13)
Mostly dissatisfied	4 (27)	3 (20)
Completely dissatisfied	5 (33)	4 (27)
Advertised role for dietitian with >10 years' experience (*n* = 7; 11%)		
Completely satisfied	2 (29)	3 (43)
Mostly satisfied	‐	‐
Somewhat satisfied	‐	‐
Somewhat dissatisfied	1 (14)	1 (14)
Mostly dissatisfied	1 (14)	‐
Completely dissatisfied	3 (43)	3 (43)

## 4 DISCUSSION

This study developed a survey and associated processes for the ongoing and timely capture of workforce and business data about private practice dietetics. Direct recruitment via business owners achieved a comparable response to the most recent dietetics workforce study,^17^ but resulted in a higher proportion of business owners compared to employed and contracted dietitians. The mean time worked in private practice was 26 hours per week, with half of participants relying solely on income derived from their work in private practice. Most participants saw themselves working in private practice long term.

Current workforce and business data about dietitians in private practice is lacking, despite data being available for other professions.^30^ The National Health Workforce Dataset, which contains workforce data about registered health practitioners in Australia as captured by the Australian Health Practitioner Regulatory Agency as part of the annual registration process,^30^, ^31^ does not include dietetics data because the profession is not regulated by the agency. Instead, the profession is regulated by Dietitians Australia, which does not capture and publish comparable workforce data. Beyond minimum workforce data, professional bodies may also play a role in capturing and reporting on more comprehensive data describing broader workforce experiences, as demonstrated by the Royal Australian College of General Practitioners, which publishes an annual report–called *Health of the Nation*–that provides a snapshot of general practice in Australia.^21^ Since 2017, the report has been used to raise awareness of current issues experienced by the general practice workforce and inform policy and decision making.^21^, ^32^ Given the importance of such data to advocacy and workforce planning,^14^, ^16^ combined with the absence of existing mechanisms that capture and report such data, there is an opportunity for Dietitians Australia to take a leadership role in capturing and reporting this data. Thus, Dietitians Australia should consider periodically capturing this data, such as during the annual credentialling process undertaken by its members.

Workforce data from this baseline survey provides the first update to private practice dietetics since Ball et al. in 2013.^17^ Several key points of divergence were observed in the current study that potentially identify the maturing of private practice as a legitimate career path for dietitians. The first point of divergence was in terms of time spent in private practice. The mean time spent in private practice was 26 hours per week, which is greater than the 68% of participants who worked less than 20 hours per week in Ball et al.^17^ and the one quarter who worked more than 20 hours in Cant et al.^19^ In addition, half of participants in Ball et al. worked in other dietetic roles to supplement their work in private practice; this proportion was lower in the current study, with more than half relying solely on income derived from their work in a single private practice. However, the most marked difference was the proportion of participants who saw themselves working in private practice long term, with 52% of respondents in Ball et al. versus 88% in the current study. Despite these findings, employment in clinical settings (48.4%) was still more preferred to private practice (33.2%) among graduates,^7^ highlighting the persistent challenge of attracting and retaining top talent, an observation supported by more than half of business owners who were dissatisfied with the number and quality of applications received for advertised roles. This may be due to neglected placement opportunities in private practice during university education, with exposure seen as a way to demonstrate the opportunities available in private practice.^12^ While private practice may be increasingly perceived as a legitimate career path for dietitians, further work is required to expose dietetics students to private practice, thereby encouraging graduate dietitians to pursue private practice as a first option.

This study suggests that dietitians derive a sense of accomplishment and satisfaction from their work in private practice, but that resilience is critical—a sentiment similarly described by experienced private practice dietitians in Donelly et al.^12^ While most dietitians felt a sense of accomplishment and satisfaction with their work, a finding that is consistent with earlier dietetics research,^10^ significant associations were observed suggesting that, without appropriate support, younger and recent graduate dietitians may be at risk of occupational fatigue or burnout leading to greater workforce attrition.^33^ The need for support has been raised previously, with recent work identifying that dietitians felt unprepared for work in private practice, highlighting the need for greater education and support for both pre‐credentialed and post‐credentialed dietitians.^12^, ^34^, ^35^ The recent Scope of Practice Review addressed the need for ‘work‐ready’ graduates by recommending relevant, practical, and high‐quality pre‐registration and post‐registration experiences in primary care.^14^ Business owners in the current study identified support and supervision of employees as a challenge within the financial constraints of primary care, highlighting the need for financial support to make such experiences possible in practice.

Several strengths and limitations of this study are noted. The recruitment strategy, with its focus on directly reaching dietitians operating a private practice, was a strength in terms of capturing input from one in four Australian dietetic practices. However, the recruitment strategy was also a limitation in terms of broader workforce participation as it relied on business owners sharing study information with employees and contractors. Thus, the response from employed and contracted dietitians was lower than anticipated, as was the response from younger dietitians, indicating that these results may not be generalisable to the broader private practice dietetics workforce. There is an opportunity to enhance broader workforce engagement by exploring other strategies, such as integration of data collection with the annual credential and membership renewal process administered by Dietitians Australia.^36^

This study provides a critical description of the private dietetic practice workforce in Australia and establishes the processes for periodic administration of the survey. Ongoing capture of data about the dietetics workforce and businesses is essential to support advocacy efforts to ensure that private practice is a legitimate and sustainable career path capable of recruiting and retaining top talent, thereby future proofing this segment of the workforce and ensuring access to high‐quality preventative and primary dietetic care.

## AUTHOR CONTRIBUTIONS

All authors contributed to the conceptualisation and design of the study. AK collected and analysed data. All authors contributed to the interpretation of results and to writing the manuscript. All authors critically reviewed the manuscript and approved the final version submitted for publication. The authors would like to thank the dietitians and other health professionals who participated in this study. We acknowledge the time‐limited nature of private practice and are incredibly grateful that you chose to spend your time participating in this study. We also acknowledge Dr. Kate Newman for her assistance with the statistical analysis.

## FUNDING INFORMATION

Professor Ball and Dr. Kirkegaard were supported by an NHMRC Investigator Grant (Grant/Award Number: APP1173496). All researchers were supported by the UQ Centre for Community Health and Wellbeing, which is part‐funded by Springfield City Group.

## CONFLICT OF INTEREST STATEMENT

Amy Kirkegaard is an Associate Editor at Nutrition & Dietetics. She was excluded from the peer‐review process and all decision making regarding this article. This manuscript has been managed throughout the review process by the Journal's Editor‐in‐Chief. The Journal operates a blinded peer review process and the peer reviewers for this manuscript were unaware of the authors of the manuscript. This process prevents authors who also hold an editorial role to influence the editorial decisions made. All other authors declare no conflicts of interest.

## ETHICS STATEMENT

This study was approved by The University of Queensland Human Research Ethics Committee (Ref No: 2023/HE001399).

## Supporting information


**TABLE S1.** Statistical analyses of ordinal variables conducted in this study using Kendall's Tau‐b.

## Data Availability

The data that support the findings of this study are available from the corresponding author upon reasonable request.
