# Supplementary material for: Development and baseline findings of a national dataset describing Australian private practice dietetics
Source: Nutr Diet. 2025 Aug 6;83(2):191–9. doi: 10.1111/1747-0080.70027 (PMC13096675; doi:10.1111/1747-0080.70027)
Supplement: Supplementary file 1 — TABLE S1. Statistical analyses of ordinal variables conducted in this study using Kendall's Tau‐b. [file NDI-83-191-s001.docx]

**Table S1.** Statistical analyses of ordinal variables conducted in this study using Kendall’s Tau-b

| **Variable 1** | **Variable 2** |
| --- | --- |
| Time since graduation | Agreement with accomplishment statement |
| Time since graduation | Agreement with remuneration statement |
| Time since graduation | Agreement with support statement |
| Time since graduation | Agreement with unwell statement |
| Time since graduation | Overall satisfaction |
| Time since graduation | Likelihood of working in private practice in 5 years |
| Time since graduation | Time spent on client activities |
| Time since graduation | Time spent on business activities |
| Age | Agreement with accomplishment statement |
| Age | Agreement with remuneration statement |
| Age | Agreement with support statement |
| Age | Agreement with unwell statement |
| Age | Overall satisfaction |
| Age | Likelihood of working in private practice in 5 years |
| Time spent on client activities | Overall satisfaction |
| Time spent on business activities | Overall satisfaction |
| Overall satisfaction | Likelihood of working in private practice in 5 years |
| Agreement with accomplishment statement | Likelihood of working in private practice in 5 years |
| Agreement with accomplishment statement | Overall satisfaction |
| Agreement with remuneration statement | Likelihood of working in private practice in 5 years |
| Agreement with remuneration statement | Overall satisfaction |
| Agreement with support statement | Likelihood of working in private practice in 5 years |
| Agreement with support statement | Overall satisfaction |
| Agreement with unwell statement | Likelihood of working in private practice in 5 years |
| Agreement with unwell statement | Overall satisfaction |
| Geographic location of practice | Satisfaction with number of applications for dietetic roles with less than 2 years’ experience |
| Geographic location of practice | Satisfaction with number of applications for dietetic roles with 2 to 5 years’ experience |
| Geographic location of practice | Satisfaction with number of applications for dietetic roles with 5 to 10 years’ experience |
| Geographic location of practice | Satisfaction with number of applications for dietetic roles with more than 10 years’ experience |
| Geographic location of practice | Satisfaction with quality of applications for dietetic roles with less than 2 years’ experience |
| Geographic location of practice | Satisfaction with quality of applications for dietetic roles with 2 to 5 years’ experience |
| Geographic location of practice | Satisfaction with quality of applications for dietetic roles with 5 to 10 years’ experience |
| Geographic location of practice | Satisfaction with quality of applications for dietetic roles with more than 10 years’ experience |
| Regionality of practice | Satisfaction with number of applications for dietetic roles with less than 2 years’ experience |
| Regionality of practice | Satisfaction with number of applications for dietetic roles with 2 to 5 years’ experience |
| Regionality of practice | Satisfaction with number of applications for dietetic roles with 5 to 10 years’ experience |
| Regionality of practice | Satisfaction with number of applications for dietetic roles with more than 10 years’ experience |
| Regionality of practice | Satisfaction with quality of applications for dietetic roles with less than 2 years’ experience |
| Regionality of practice | Satisfaction with quality of applications for dietetic roles with 2 to 5 years’ experience |
| Regionality of practice | Satisfaction with quality of applications for dietetic roles with 5 to 10 years’ experience |
| Regionality of practice | Satisfaction with quality of applications for dietetic roles with more than 10 years’ experience |
